# Supplementary material for: PopDel identifies medium-size deletions simultaneously in tens of thousands of genomes
Source: Nat Commun. 2021 Feb 1;12:730. doi: 10.1038/s41467-020-20850-5 (PMC7851401; doi:10.1038/s41467-020-20850-5)
Supplement: Supplementary file 1 — Supplementary Information [file 41467_2020_20850_MOESM1_ESM.pdf]

Supplementary Information:

PopDel identifies medium-size deletions simultaneously in tens of thousands of genomes

Sebastian Niehus[1,2,3], Hákon Jónsson[4], Janina Schönberger[2,3], Eythór Björnsson[4,5,6], Doruk Beyter[4], Hannes P. Eggertsson[4,] Patrick Sulem[4], Kári Stefansson[4,5], Bjarni V. Halldórsson[4,7], Birte Kehr[1,2,3,8]

[1] Regensburg Center for Interventional Immunology (RCI), Franz-Josef-Strauss-Allee 11, 93053 Regensburg, Germany

[2] Berlin Institute of Health (BIH) 10178 Berlin, Germany

[3] Charité – Universitätsmedizin Berlin, corporate member of Freie Universität Berlin, Humbold-Universität zu Berlin, and Berlin Institute of Health

[4] deCODE genetics/Amgen Inc.

[5] Faculty of Medicine, School of Health Sciences, University of Iceland

[6] Department of Internal Medicine, Landspítali – The National University Hospital of Iceland, Reykjavík, Iceland

[7] School of Science and Engineering, Reykjavik University

[8] Faculty of Medicine, University of Regensburg, 93053 Regensburg, Germany

Table of Contents

Supplementary Tables 5

Supplementary Figures 13

Supplementary Notes 17

Supplementary Note 1: Implementation details of PopDel 17

1.1. Binary profile format 17

1.2. Buffering and length limitations during profile creation 19

1.3. Sampling regions for creating the insert size histogram 19

1.4. Robust estimation of median and standard deviation of insert size histogram 19

1.5. Note on representations of numeric values during the calling 19

1.6. Handling of multiple read groups in a sample 20

1.7. Initialization of the deletion length and allele frequency 21

1.8. Transformation of insert size histogram 21

1.9. Reference shift 22

1.10. Termination of iterative parameter estimation 22

1.11. Deletion start position estimation 23

1.12. Conditions for the combination of consecutive windows 24

1.13. Additional filters during joint calling 25

Supplementary Note 2: Evaluation on simulated and real data 25

2.1. Simulated data with random deletions 25

2.2. Simulated 1000 Genomes Project data 29

2.3. Evaluation of single and trio wise calling using NA12878 and the HG002 trio 30

2.4. Evaluation on the Polaris Diversity and Kids cohort 34

2.5. Evaluation on Icelandic data 36

References 40

Supplementary Tables

Supplementary Table 1 - Alignment requirements in PopDel's profiling step. 5

Supplementary Table 2 - Human readable representation of PopDel's profile format. 6

Supplementary Table 3 – True positive calls on simulation with random deletions in human chromosome 21.. 7

Supplementary Table 4 - False positive calls on simulation with random deletions in human chromosome 21.. 8

Supplementary Table 5 - False negatives on simulation with random deletions in human chromosome 21.. 9

Supplementary Table 6 – True positive calls on simulation with 1000 Genomes Project deletions in chromosomes 17 to 22. 10

Supplementary Table 7 - False positive calls on simulation with 1000 Genomes Project deletions in chromosomes 17 to 22. 11

Supplementary Table 8 - False negatives on simulation with 1000 Genomes Project deletions in chromosomes 17 to 22. 12

Supplementary Table 9 - Definition of PopDel's binary profile format. 18

Supplementary Figures

Supplementary Figure 4 - Venn diagrams of call set overlaps on NA12878..........................13

Supplementary Figure 5 - Venn diagrams of call set overlaps on HG002. 14

Supplementary Figure 6 – PCA biplot for **a** Delly and **b** Lumpy**.** 15

Supplementary Figure 1 – Visualization of Mendelian consistency. 15

Supplementary Figure 2 - Visualization of the parts of a read pair and its insert size............16

Supplementary Figure 3 - Disk space used by PopDel's profile files...................................16

Supplementary Figure 7 – Histograms of deletion start position deviations. 24

Supplementary Figure 8 – Genotyping evaluation based on alternative overlap evaluation. .28

Supplementary Figure 9 – PopDel's resource consumption for varying buffer sizes. 29

Supplementary Figure 10 - Overview of the overlap generation workflow.............................33

Supplementary Figure 11 - Mendelian inheritance error rate on HG002 trio. 34

Supplementary Figure 12 – IGV screenshot showing a rare deletion overlapped by a longer common deletion. 38

Supplementary Figure 13 – Distribution of deletions per genome as identified jointly by PopDel on 4,959 Icelandic genomes (n=168,101 deletions). 39

Supplementary Figure 14 – Histogram of deletions lengths identified jointly by PopDel on 4,959 Icelandic genomes (n=168,101 deletions). 39

Supplementary Figure 15 – Histogram of allele frequencies of deletions identified jointly by PopDel on 4,959 Icelandic genomes (n=168,101 deletions). 40

Supplementary Tables

Supplementary Table 1 - Alignment requirements in PopDel's profiling step.

Parameter	Description	Default*
orientation	The two reads of the pair align in forward-reverse orientation.	true
unclipped	Minimum number of aligned base pairs per read.	50
mapping-qual	Minimum value of the mapping quality per read.	1
align-score	Minimum alignment score per read (in percent of the length of the aligned part of the read).	80
flags-unset	SAM flags that are not set in both reads	3840

* All parameters are user configurable.

Only read pairs that meet all of the listed requirements in both reads are included in PopDel's profiles.

Supplementary Table 2 - Human readable representation of PopDel’s profile format.

Chromosome	Window position	Read pair information
chr21	5035777	230:-10, 233:-34
chr21	5036033	3:-37, 11:0, 19:-3, 214:15
chr21	5036289	4:-85, 4:-29, 13:-96, 13:-78:17, 80:-5
chr21	5036545	172:5, 178:51, 180:13, 192:19, 248:-58
chr21	5036801	51:35, 62:-50, 83:-60, 85:33, 110:88, 218:104
chr21	5037057	196:53, 210:12, 210:43, 232:-77, 253:-19
chr21	5037313	11:51, 15:-63, 27:77, 44:10, 64:-73, 65:-88

PopDel’s profile format is divided into windows of 256 bp. The start position of each window is given in column one and two. The information on read pairs that fall in the respective window is given in column three, divided by ‘,’. The number before the ‘:’ indicates the offset of the start position from the start of the window and the value after the ‘:’ is the deviation from the median insert size μ. The start position of a pair refers to the rightmost aligned base of the forward read of a pair. Each read pair is only listed in the window that contains its start position.

Supplementary Table 3 – True positive calls on simulation with random deletions in human chromosome 21.

Samples	PopDel	Delly	Manta	Lumpy	GRIDSS
1	1157	**1166**	1145	1140	1148
2	**1414**	1408	1395	1380	1396
3	**1530**	1517	1509	1491	1515
4	**1587**	1583	1572	1553	1579
5	**1633**	1630	1616	1596	1625
6	**1652**	1645	1633	1611	1643
7	**1672**	1664	1652	1629	1663
8	**1690**	1678	1666	1643	1679
9	**1696**	1683	1671	1648	1685
10	**1702**	1687	1676	1653	1690
20	**1748**	1732	1723	1702	1736
30	**1766**	1748	1739	1718	1749
40	**1775**	1757	1746	1728	1756
50	**1781**	1763	1754	1734	1764
60	**1783**	1765	1755	1737	1767
70	**1787**	1767	1758	1739	1771
80	**1789**	1769	1759	1740	1771
90	**1790**	1770	1761	1741	1770
100	**1790**	1771	1762	1742	1772
200	**1794**	1777	1761	1748	1773
300	**1794**	1782	1761	1750	1777
400	**1795**	1785	1764	1751	1779
500	**1795**	1787	1763	1751	NA
600	**1793**	1790	1764	1753	NA
700	**1795**	1792	1765	1755	NA
800	**1795**	1793	1765	1755	NA
900	**1795**	1794	1762	1755	NA
1000	**1794**	1793	1762	1756	NA

The maximum value of each row is highlighted in bold font.

Supplementary Table 4 - False positive calls on simulation with random deletions in human chromosome 21.

Samples	PopDel	Delly	Manta	Lumpy	GRIDSS
1	**0**	**0**	**0**	**0**	0
2	**0**	**0**	**0**	**0**	0
3	**0**	**0**	**0**	**0**	0
4	**0**	**0**	**0**	**0**	0
5	**0**	**0**	**0**	**0**	0
6	**0**	**0**	**0**	**0**	0
7	**0**	**0**	**0**	**0**	0
8	**0**	**0**	1	**0**	0
9	**0**	**0**	1	**0**	0
10	**0**	**0**	1	**0**	0
20	**0**	**0**	**0**	**0**	0
30	**0**	**0**	**0**	**0**	0
40	1	**0**	1	**0**	0
50	1	**0**	1	**0**	5
60	**0**	**0**	1	**0**	9
70	**0**	**0**	1	**0**	17
80	**0**	**0**	1	**0**	29
90	**0**	1	1	**0**	58
100	1	1	1	**0**	69
200	**0**	2	4	**0**	82
300	1	3	4	**0**	134
400	**0**	5	4	**0**	309
500	1	5	3	**0**	NA
600	2	6	4	**0**	NA
700	1	8	4	**0**	NA
800	1	11	4	**0**	NA
900	1	13	4	**0**	NA
1000	1	13	4	**0**	NA

The minimum values of each row are highlighted in bold font.

Supplementary Table 5 - False negatives on simulation with random deletions in human chromosome 21.

Samples	PopDel	Delly	Manta	Lumpy	GRIDSS
1	159	**150**	171	176	168
2	**185**	191	204	219	203
3	**189**	202	210	228	204
4	**204**	208	219	238	212
5	**205**	208	222	242	213
6	**203**	210	222	244	212
7	**202**	210	222	245	211
8	**201**	213	225	248	212
9	**200**	213	225	248	211
10	**198**	213	224	247	210
20	**203**	219	228	249	215
30	**197**	215	224	245	214
40	**197**	215	226	244	216
50	**198**	216	225	245	215
60	**199**	217	227	245	215
70	**197**	217	226	245	213
80	**198**	218	228	247	216
90	**198**	218	227	247	218
100	**199**	218	227	247	217
200	**199**	216	232	245	220
300	**202**	214	235	246	219
400	**203**	213	234	247	219
500	**203**	211	235	247	NA
600	**205**	208	234	245	NA
700	**204**	207	234	244	NA
800	**204**	206	234	244	NA
900	**204**	205	237	244	NA
1000	**206**	207	238	244	NA

The minimum values of each row are highlighted in bold font.

Supplementary Table 6 – True positive calls on simulation with 1000 Genomes Project deletions in chromosomes 17 to 22.

Samples	PopDel	Delly	Manta	Lumpy
1	176	163	178	**192**
2	290	262	287	**303**
3	379	343	369	**388**
4	417	378	410	**427**
5	456	408	456	**470**
6	491	441	492	**505**
7	520	466	528	**537**
8	541	483	551	**559**
9	558	502	569	**581**
10	582	524	592	**604**
20	773	717	790	**803**
30	913	855	938	**952**
40	999	948	1025	**1042**
50	1085	1040	1108	**1127**
60	1159	1126	1193	**1215**
70	1272	1248	1304	**1331**
80	1339	1313	1371	**1400**
90	1401	1371	1435	**1464**
100	1466	1443	1502	**1534**
200	2048	2029	2088	**2138**
300	2452	2431	2442	**2559**
400	2721	2699	2639	**2846**
500	2962	2944	2856	**3111**

The maximum value of each row is highlighted in bold font.

Supplementary Table 7 - False positive calls on simulation with 1000 Genomes Project deletions in chromosomes 17 to 22.

Samples	PopDel	Delly	Manta	Lumpy
1	**0**	**0**	**0**	**0**
2	**0**	1	**0**	**0**
3	**0**	1	1	**0**
4	**0**	1	1	**0**
5	**0**	1	1	**0**
6	**0**	1	1	**0**
7	**0**	1	1	1
8	**0**	1	1	1
9	**1**	**1**	**1**	**1**
10	2	**1**	**1**	**1**
20	2	1	7	**0**
30	5	1	8	**0**
40	4	2	9	**0**
50	4	1	8	**0**
60	5	3	12	**0**
70	9	3	13	**0**
80	8	3	15	**0**
90	8	4	16	**0**
100	8	4	16	**0**
200	13	10	18	**0**
300	16	13	20	**0**
400	13	17	24	**0**
500	15	26	28	**0**

The minimum values of each row are highlighted in bold font.

Supplementary Table 8 - False negatives on simulation with 1000 Genomes Project deletions in chromosomes 17 to 22.

Samples	PopDel	Delly	Manta	Lumpy
1	35	48	33	**19**
2	42	70	45	**29**
3	46	82	56	**37**
4	48	87	55	**38**
5	47	95	47	**33**
6	47	97	46	**33**
7	51	105	43	**34**
8	54	112	44	**36**
9	59	115	48	**36**
10	60	118	50	**38**
20	86	142	69	**56**
30	100	158	75	**61**
40	110	161	84	**67**
50	112	157	89	**70**
60	129	162	95	**73**
70	138	162	106	**79**
80	140	166	108	**79**
90	144	174	110	**81**
100	150	173	114	**82**
200	202	221	162	**112**
300	239	260	249	**132**
400	255	277	337	**130**
500	284	302	390	**135**

The minimum value of each row is highlighted in bold font.

Supplementary Figures

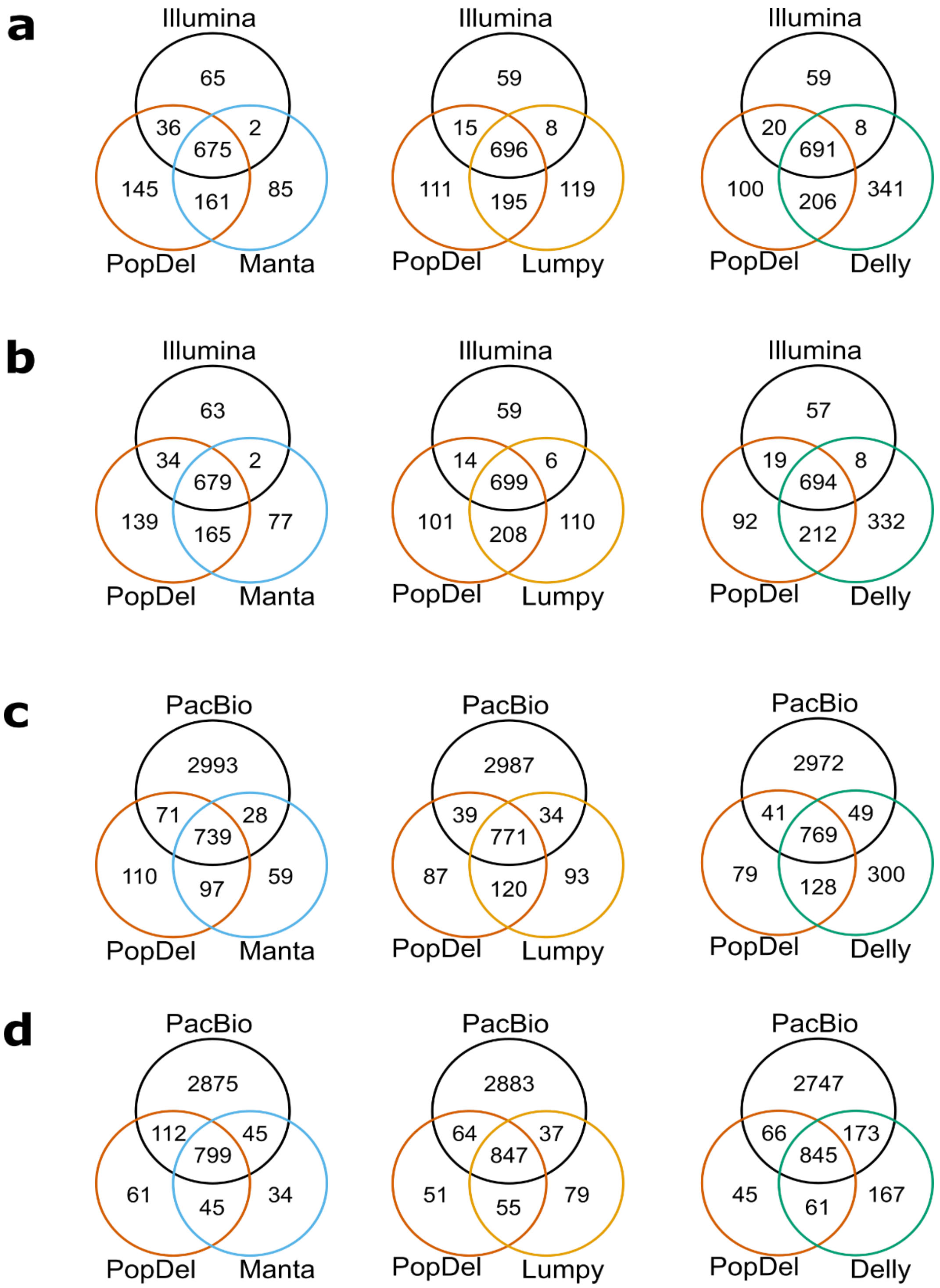

Supplementary Figure 1 - Venn diagrams of call set overlaps on NA12878. **a, b** Comparison with Illumina short read reference set. **c, d** Comparison with PacBio long read reference set. **a, c** Comparison using a minimum reciprocal overlap of 80%. **b, d** Comparison using a minimum reciprocal overlap of 0.1%.

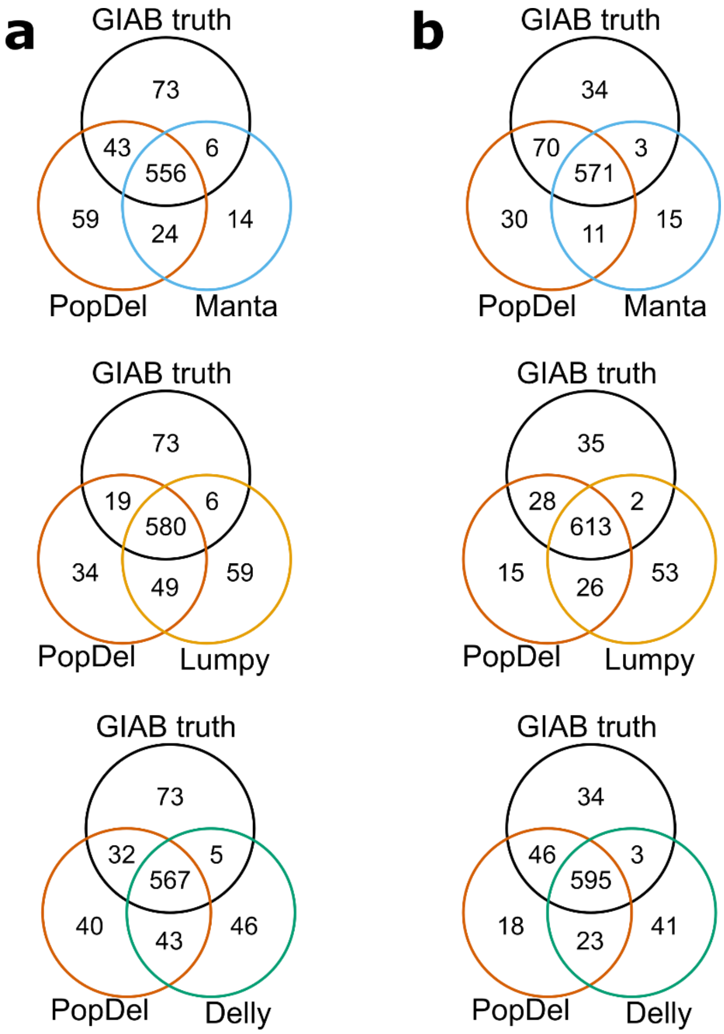

Supplementary Figure 2 - Venn diagrams of call set overlaps on HG002. Comparison with GIAB truth set in high confidence regions for minimum reciprocal overlap of **a** 80% and **b** 0.1%.

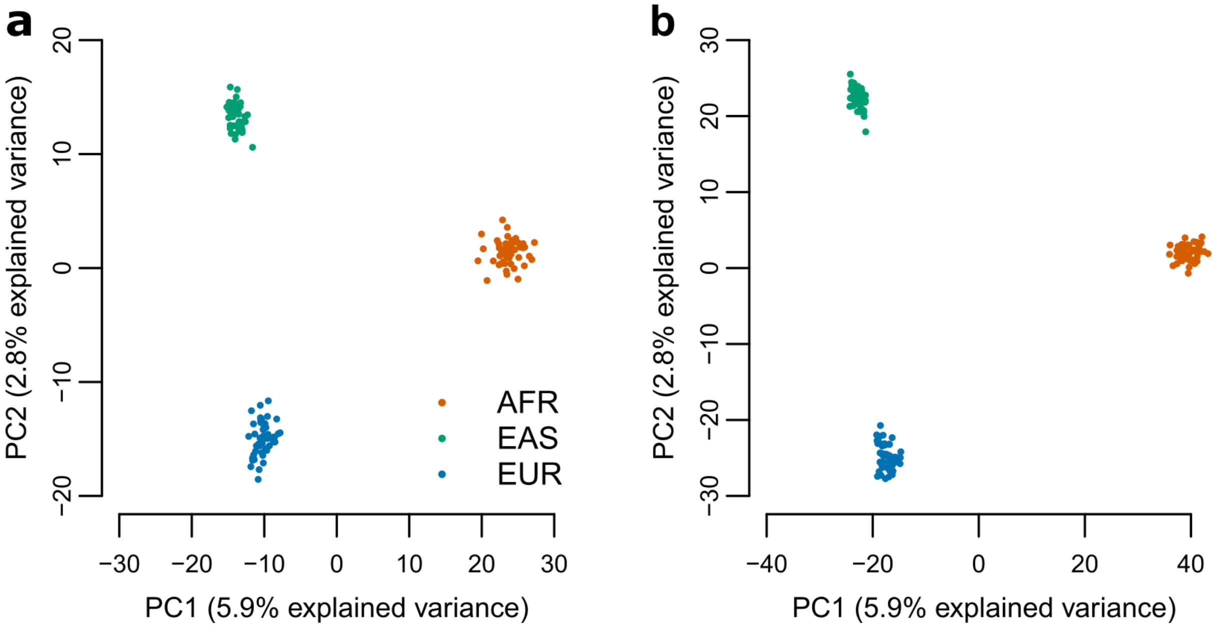

Supplementary Figure 3 – PCA biplot for **a** Delly and **b** Lumpy**.** PCA calculations were based on the variant allele counts of the tools for the Polaris Diversity cohort. AFR, African; EAS, East Asian; EUR, European.

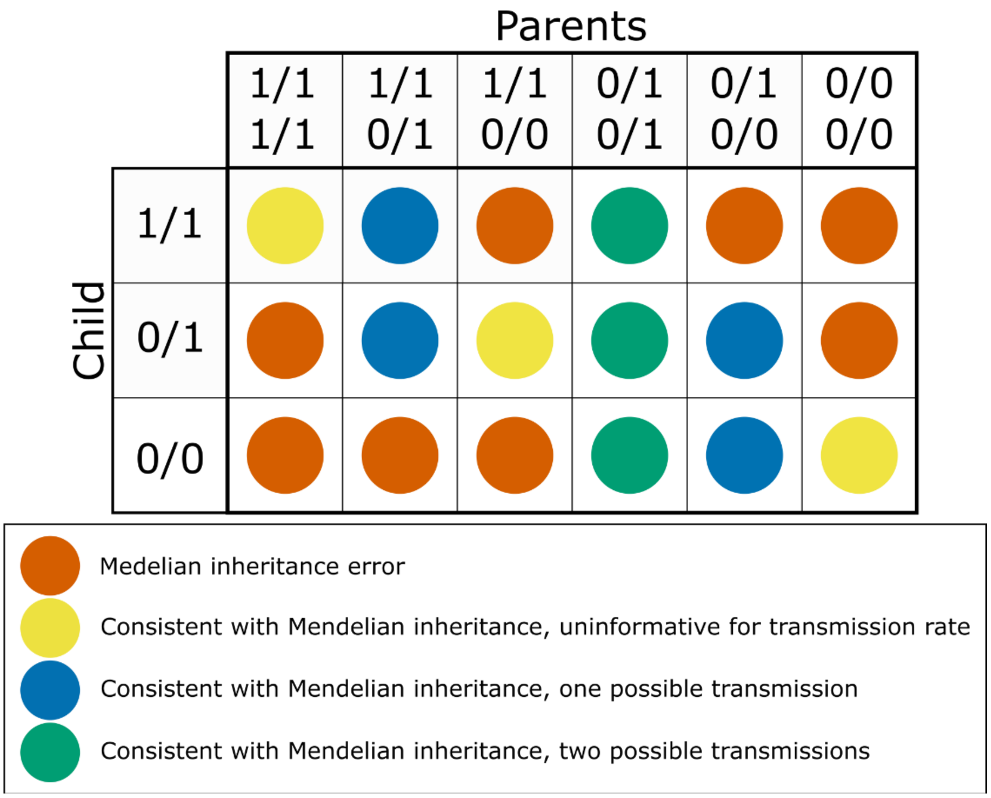

Supplementary Figure 4 – Visualization of Mendelian consistency. The columns indicate the genotype combinations of the parents while the rows indicate the genotypes of the child. The colors indicate whether the genotype of the child is consistent with the rules of Mendelian inheritance and whether it is informative for calculating the transmission rate or not.

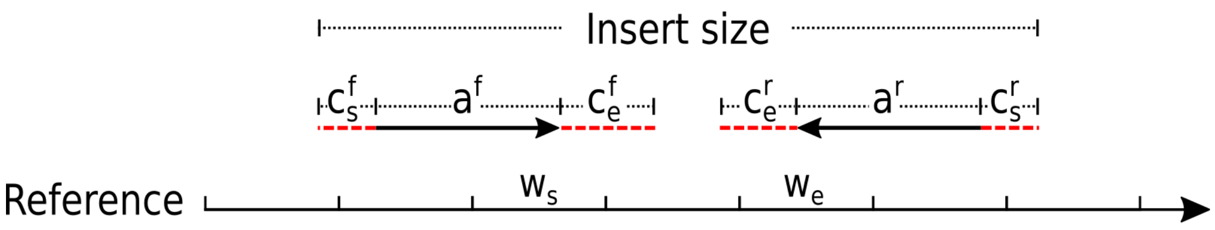

Supplementary Figure 5 - Visualization of the parts of a read pair and its insert size. The insert size includes the length of the aligned parts of the reads (a^f, a^r) plus the outer soft clipped bases (c_s^f, c_s^r). The starting window w_s is the window on the reference where the right end of a^f aligns. The end window w_e is the window where the left end of a^r aligns. All windows between w_s and w_e (inclusive) are considered to be overlapped by the read pair.

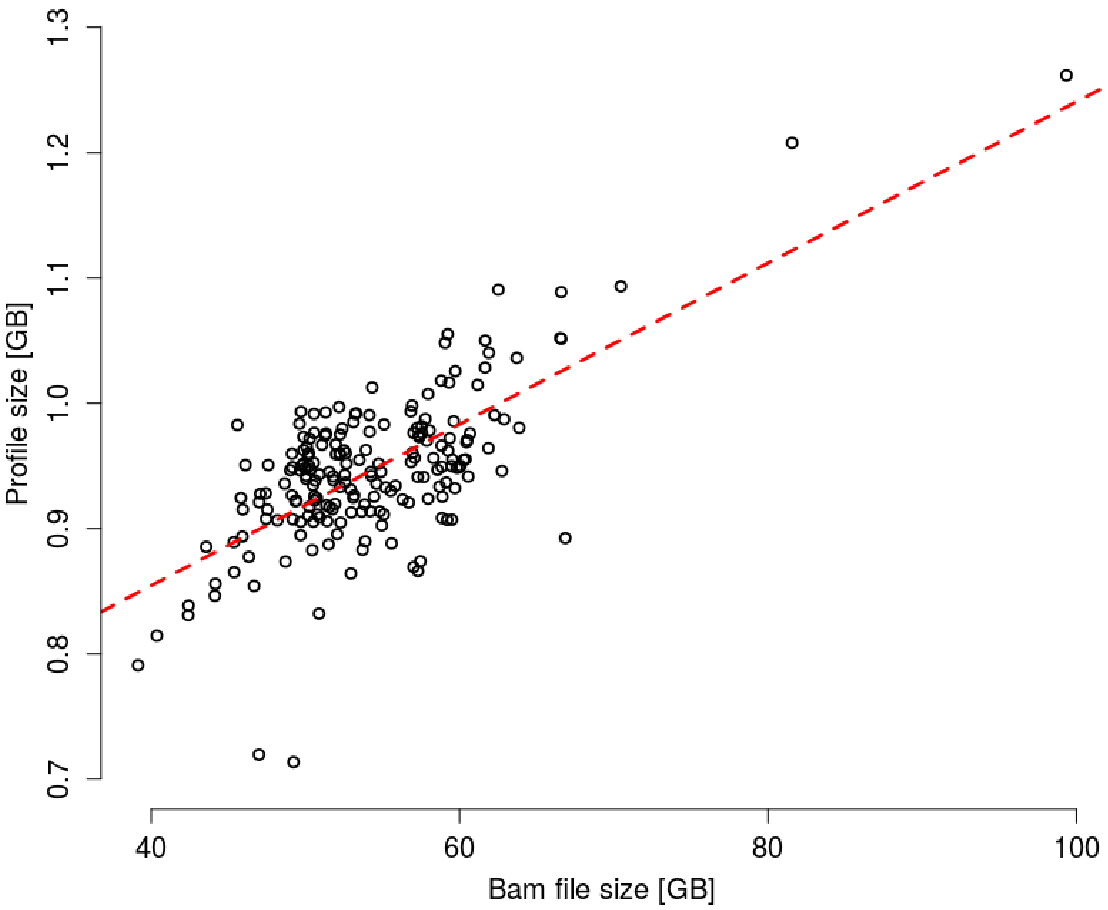

Supplementary Figure 6 - Disk space used by PopDel's profile files. Comparison of the disk space used by of PopDel's profile files to the disk space used by the original BAM files of all Polaris (Diversity and Kids cohort) samples. The red line indicates a linear regression line. The median profile size is 0.9 GB, which is 1.78% of the median BAM file size of 53.1 GB. The mean relative size of the profiles is 1.76%.

Supplementary Notes

Supplementary Note 1: Implementation details of PopDel

1.1. Binary profile format

The binary format, defined in Supplementary Table 9, stores insert sizes efficiently on disk. One important factor is avoiding the suboptimal representation of big integers as strings of chars (assuming 8 bits per char, a number consisting of e.g. 7 digits, such as genomic coordinates, require $7 \cdot 8 = 56$ bits of space; using a 32-bit unsigned integer instead, PopDel can store the same number in 24 fewer bits). Further disk space is saved by avoiding redundant information like the full name of the chromosome for each genomic position. Instead, a 32-bit unsigned integer is used as identifier. Thanks to the strict size definition of every entry, it is possible to avoid separators like whitespaces, commas, or colons otherwise necessary. This also enables efficient indexing and jumping in the file. Lastly, the stored insert sizes are grouped in genomic windows of 256 base pairs based on their start positions, as can be seen in Supplementary Table 2. This way the positional information has to be stored only once per window and not per insert size. The offset of each read pair's position from the start position of the window is stored as a single char (which is why we use windows of size 256). If one wants to inspect the profile, PopDel offers the function 'view' to convert the profile into a human readable format.

Supplementary Table 9 - Definition of PopDel's binary profile format.

Field	Description	Type	Value
magic	PopDel magic string	char[7]	POPDEL\1
l_region	Index region size	uint32_t	
n_file_offsets	# file offsets in the index (index size)	uint32_t	
List of file offsets (n=n_file_offsets)			
file_offset	File offset (index entry)	uint64_t	
n_rg	# read groups		
List of read group names and insert size histograms (n=n_rg)			
l_rg_name	Length of the read group name plus 1 (including NUL)	uint32_t	
rg_name	Read group name; NUL-terminated	char[l_rg_name]	
median_isize	Median insert size in this read group	int16_t	
stddev_isize	Standard deviation of insert size in this read group	int16_t	
read_length	Length of reads in this read group	uint16_t	
hist_start	First insert size listed in histogram	uint16_t	
hist_end	Last insert size listed in histogram plus 1	uint16_t	
List of read pair counts per insert size (n=hist_end - hist_start); insert size histogram			
count	# read pairs with corresponding insert size	uint32_t	
n_ref	# reference sequences	uint32_t	
List of contig names (n=n_ref)			
l_ref_name	Length of the reference name plus 1 (including NUL)	uint32_t	
ref_name	Reference sequence name; NUL-terminated	char[l_ref_name]	
l_ref	Length of the reference sequence	uint32_t	
List of windows (until the end of the file)			
refID	Reference sequence ID, 0 ≤ refID < n_ref	uint32_t	
pos	Start position of the window (0-based)	uint32_t	
List of read group entries for the window (n=n_rg)			
n_rp	# read pairs	uint32_t	
List of read pairs for the read group in the window (n=n_rp)			
pos_offset	Offset of read pair position from window start position	char	
isize_dev	Deviation from median insert size	int16_t	

The first column describes the name of the field, whose content is described in the second column. Column 3 indicates the datatype and the size of the entry. Except for the magic string, which is used for identifying the profile format and version, no field has default value as indicated by column 4. A line saying "List of …(n=n_x)" indicates that the following block is repeated n times, potentially with different content.

1.2. Buffering and length limitations during profile creation

PopDel creates the profile by checking the reads of the BAM file one after the other. If a read is in the forward orientation and its corresponding reverse read maps at most $l_{\max} + \mu_S$ base pairs downstream (with $l_{\max}$ as the maximum deletion length, which defaults to 10,000 bp, and μ_S as the median of the sample's insert sizes), the read is added to a buffer. If a read is in reverse orientation, the corresponding forward read is removed from the buffer and the pair's insert size is added to the profile. This buffering ensures that both reads fulfil the requirements listed in Supplementary Table 1 without jumping in the BAM file. The default value of 10,000 bp for $l_{\max}$ has been selected to keep the buffering to a minimum and because we argue that deletions above a size of 10,000 bp can be easily found using read depth methods. This value can be increased by the user.

1.3. Sampling regions for creating the insert size histogram

The sampling uses a set of 22 default regions, one 1,000,000 bp region from each autosome. The regions can also be specified by the user. PopDel parses all reads passing the filters in the first of the desired regions and counts the number of read pairs per insert size to obtain an empirical histogram of the insert sizes. If the minimum number of sampled read pairs (default 50,000 per read group) has been reached, no further regions are used for sampling. Otherwise, the sampling continues with the next regions. The regions are listed in PopDel's wiki on GitHub.

1.4. Robust estimation of median and standard deviation of insert size histogram

The median μ_S and standard deviation σ_S of the insert size distribution are estimated and refined by trimming the histogram to the insert size interval $[\mu_S - 3\sigma_S, \mu_S + 3\sigma_S]$, then repeating the calculation and trimming again until both μ_S and σ_S converge. The final histogram is called H_{abs}^S. The refinement mitigates the effect outliers when calculating μ_S and σ_S, contributing to the overall robustness of the estimation.

1.5. Note on representations of numeric values during the calling

Due to the potentially large amount of data in a single window and the numerous multiplications of very small likelihoods, it is to be expected that numbers might occasionally drop below the smallest possible values for the data type double. To avoid this, the values of the histograms are stored as their log-values and all likelihood ratios are internally computed as log-likelihood-ratios. This results in an addition of the log-likelihood ratios instead of multiplications and a bigger domain for the possible values. The formulas in the Methods and Supplementary text nevertheless avoid the logarithms to keep the underlying formulas as concise as possible.

1.6. Handling of multiple read groups in a sample

For some experiments, a sample might consist of multiple read groups, which further might originate from different libraries with distinct insert size distributions. PopDel handles this by creating one insert size histogram per read group instead of per sample. During the calling, PopDel handles all reads according to the read group they originate from, i.e. it compares the insert sizes to the corresponding distribution. All insert size histogram related parameters ($H(x), \mu, \sigma$) as well as the reference shift ϵ and the weights a_g are calculated and handled on a per read group basis.

For the calculation of the likelihoods, we obtain:

$$\mathcal{L}(\text{no del}) = \pi \prod_{S \in \mathcal{S}} \prod_{R \in \mathcal{R}^S} \mathcal{L}(G_0|\Delta^R) \tag{1}$$

$$\mathcal{L}(\text{del of length } l) = (1-\pi) \prod_{S \in \mathcal{S}} \prod_{R \in \mathcal{R}^S} a_0^R \cdot \mathcal{L}(G_0|\Delta^R) + a_1^R \cdot \mathcal{L}(G_1|\Delta^R) + a_2^R \cdot \mathcal{L}(G_2|\Delta^R) \tag{2}$$

with $\mathcal{R}^S$ as the set of read groups of sample S, Δ^R is the set of insert size deviations of read group R in the current window and π as the prior (default 10^{-4}). We further have:

$$\mathcal{L}(G_0|\Delta^R) = \prod_{\delta \in \Delta^R} H^R(\delta - \epsilon_R) \tag{3}$$

$$\mathcal{L}(G_1|\Delta^R) = \prod_{\delta \in \Delta^R} \frac{H^R(\delta - \epsilon_R) + H^R(\delta - l)}{2} \tag{4}$$

$$\mathcal{L}(G_2|\Delta^R) = \prod_{\delta \in \Delta^R} H^R(\delta - l) \tag{5}$$

where $H^R(x)$ denotes the transformed insert size histogram of read group R (see section 1.8). The sample and genotype specific weights become read group and genotype specific weights:

$$a_g^R = \frac{\mathcal{L}(G_g|\Delta^R) \cdot F(f, G_g)}{\sum_{j=0}^{2} \left(\mathcal{L}(G_j|\Delta^R) \cdot F(f, G_j)\right)} \tag{6}$$

For the allele frequency f we obtain:

$$f^{\text{new}} = \frac{1}{2|\mathcal{S}|} \cdot \sum_{S \in \mathcal{S}} \sum_{R \in \mathcal{R}^S} (a_1^R + 2a_2^R) \tag{7}$$

We update the deletion length estimate as:

$$l^{\text{new}} = \frac{\sum_{S \in \mathcal{S}} \sum_{R \in \mathcal{R}^S} \sum_{\delta \in \Delta^R} \delta \cdot P_{l,\epsilon_R}^R(\delta)}{\sum_{S \in \mathcal{S}} \sum_{R \in \mathcal{R}^S} \sum_{\delta \in \Delta^R} P_{l,\epsilon_R}^R(\delta)} \tag{8}$$

with

$$P^R_{l,\epsilon_R}(\delta) = a^R_1 \cdot \frac{H^R(\delta - l)}{H^R(\delta - \epsilon_R) + H^R(\delta - l)} + a^R_2 \quad (9)$$

1.7. Initialization of the deletion length and allele frequency

Given the insert size deviations of read pairs overlapping some genomic window, PopDel first suggests one initial deletion length per sample and then clusters these values across all samples into a small set of initial deletion lengths. More specifically, PopDel inspects the insert size deviations of each sample S separately and selects the third quartile $Q_3(\Delta^S)$ as an initial length suggestion. If $Q_3(\Delta^S) \geq 4\sigma_S$ it is added to a list. After processing all samples, the values on this list are clustered such that values that differ by at most 50 bp end up in the same cluster. The means of all clusters are subsequently used as initial deletion lengths l^{init}. For each l^{init} an initial allele frequency is calculated as:

$$f^{\text{init}} = \frac{\sum_{S\in\mathcal{S}} \sum_{\delta\in\Delta^S} b_{l,2\sigma_S}(\delta)}{2|\mathcal{S}|}, \quad b_{l,2\sigma_S}(\delta) = \begin{cases} 1 & \text{if } x \geq \max\left(l - 2\sigma_S, \frac{l}{2}\right) \wedge \delta \leq l + 2\sigma_S \\ 0 & \text{else} \end{cases} \quad (10)$$

where $|\mathcal{S}|$ denotes the total number of samples.

1.8. Transformation of insert size histogram

The histogram H^S_{abs}, which has been created during the profiling step, stores absolute counts of observed insert sizes for sample S. Let $H^S_{\text{abs}}(i)$ return the number of counts for an insert size i. We compute a transformed histogram:

$$H^S_{\text{trans}}(\delta) := H^S_{\text{abs}}(\delta + \mu_S) \cdot \frac{w + \max(\delta + \mu_S, 0)}{w} \quad (11)$$

Since PopDel subsequently only uses the deviation of insert sizes from the mean and not the insert sizes themselves, H^S_{trans} takes the deviation δ of the insert size as an argument.

The probability density function for the distribution is then calculated as follows:

$$H^S(\delta) := \frac{H^S_{\text{trans}}(\delta)}{\sum_j H^S_{\text{trans}}(j)} \quad (12)$$

In the cases where the sampled distribution would return zero a pseudo-count of default $\frac{\max(H^S)}{500}$ is applied instead. This is necessary to avoid a division by zero or trying to take the logarithm of zero in subsequent steps.

The reasoning behind this transformation is as follows. Since a bigger insert size and a higher window size increases the frequency of a read pair overlapping a window, we scale the values returned by $H_{\text{abs}}^{S}(i)$ with respect to insert size and window size. The number of possible mapping locations so that a read pair with insert size i and read length r overlaps the window of length w is $w + \max(i - 2r, 0)$. This is because PopDel considers a read pair to overlap a window, if any part between the right end of the forward read and the left end of the reverse read maps to the window. The reads themselves are excluded. In case the forward and reverse read overlap, PopDel takes the rightmost alignment position of the forward read as a reference point. By dividing the size of the overlap by the window length, we gain the average number of consecutive windows, a read pair with insert size i overlaps.

1.9. Reference shift

Local biases such as GC-content can result in a local shift of the insert size distribution. To avoid that those shifts result in a false positive deletion call of the window, we introduced the reference shift ϵ. To this end, we allow the subtraction of ϵ from μ_S in the calculation of the likelihood for the reference model. ϵ is updated in a similar fashion as the deletion length, however without the sum across all samples, because ϵ is specific for each individual sample:

$$\epsilon_S^{\text{new}} = \frac{\sum_{\delta \in \Delta^S} \delta \cdot P_{d,\epsilon_S}^{S}(\delta)}{\sum_{\delta \in \Delta^S} P_{d,\epsilon_S}^{S}(\delta)} \tag{13}$$

As we expect only small biases, we limit the maximum value for ϵ to the standard deviation σ_S of the sample's insert size distribution. Larger values may originate from homozygous deletions and interfere with the size estimations. Therefore, if ϵ becomes greater than σ_S, it is set to 0. Setting ϵ to σ_S instead, can distort the predicted length of homozygous deletions by this value.

1.10. Termination of iterative parameter estimation

The iteration of PopDel's window-wise calling stops if at least one of the following conditions is met:

- The new estimates for the pair of deletion length and frequency has been observed before
- The maximum number of iterations (default 15) is exceeded
- The allele frequency drops below a threshold (default 10^{-10})
- The estimate for the deletion length drops below a minimum length (default 95% quantile of $4\sigma_S$ for all $S \in \mathcal{S}$).

The two latter cases result in no call being made for the respective window for that specific deletion. In case two pairs of estimates for frequency and deletion length alternate during the iteration, PopDel assumes the pair yielding the higher likelihood ratio to be the correct one.

1.11. Deletion start position estimation

During the iteration in one window, PopDel keeps track of the positions of read pairs whose insert size deviation from the sample mean supports the deletion estimate. Let $Q_{99}(H^S)$ be the 99% quantile of the distribution of insert size deviations of sample S. A read pair overlapping the window with insert size deviation δ is considered to support a deletion of length l if $l - Q_{99}(H^S) \leq \delta \leq l + Q_{99}(H^S)$. The positions of deletion supporting read pairs are stored in a sorted list and the 80% quantile of this list is taken as the start position of the variant. This provides us with a precise estimate of the deletion start position, since the position of the read pairs stored by PopDel is the rightmost aligned position of the forward read in each pair. Therefore, PopDel implicitly considers the split-reads for estimating the precise start position.

We used our simulated data to quantify the exactness of this approach. We calculated the deviation of the tools' predicted start positions from the actual simulated position in the first 10 samples of the random deletion simulation (Methods, Supplementary text section 2.1.7). Supplementary Figure 7 shows that our approach is on par with the other tools that explicitly consider split-read alignments.

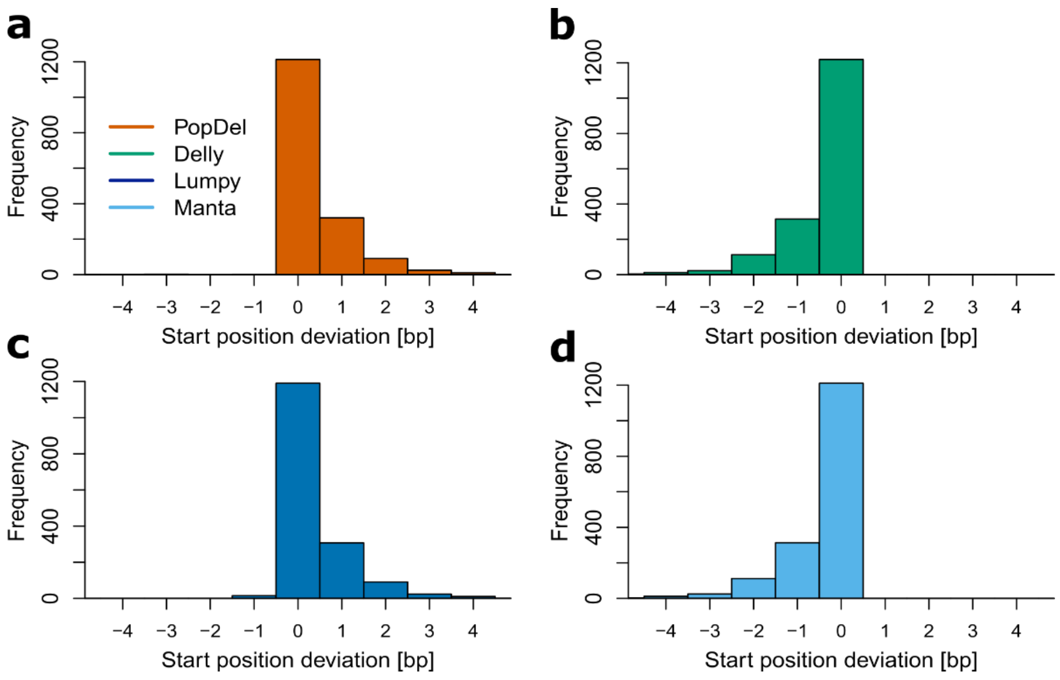

Supplementary Figure 7 – Histograms of deletion start position deviations. **a** PopDel (n=1704 predicted variants), **b** Delly (n=1686 predicated variants), **c** Lumpy (n=1653 predicted variants), **d** Manta (n=1677 predicted variants). The deviation was calculated as the difference between predicted and simulated position of the variant. A negative deviation indicates that the predicted position is upstream of the simulated position. Variants were not left or right aligned. Variants from 10 uniformly simulated chromosome 21 samples were used for the comparison.

1.12. Conditions for the combination of consecutive windows

Let w_i be the first of n windows for which the null hypothesis can be rejected and let $w_{i+k}, 0 < k < n$ be another such window. Let l_i and l_{i+k} be the deletion length estimates for w_i and w_{i+k} respectively. Let $r_i := (\mathrm{delStart}(w_i), \mathrm{delEnd}(w_i))$ denote the range spanned by the leftmost and rightmost alignment positions of the read pairs whose insert sizes support w_i and let r_{i+k} be defined analogously. When considering one window after the other, we test if the following conditions for w_i and w_{i+k} are met:

a) $|l_i - l_{i+k}| \leq \max\left(\frac{\min(l_i, l_{i+k})}{2}, 2\bar{\sigma}\right)$, with $\bar{\sigma}$ as the mean of all σ_S of all samples.

b) r_i and r_{i+k} overlap by at least $\min\left(\frac{\min(r_i, r_{i+k})}{4}, \min(r_i, r_{i+k}) - 2\bar{\sigma}\right)$.

If both a) and b) are satisfied, w_i and w_{i+k} are combined. If only a) is satisfied, an additional check is preformed to see if the deletion is interrupted:

c) $(|r_i| < l_i \lor |r_{i+k}| < l_{i+k}) \land \mathrm{delStart}(w_{i+k}) - \mathrm{delStart}(w_i) < \min(l_i, l_{i+k}) + 4\sigma$

where $|r_i|$ denotes the length of the spanned range. If a) and c) are true, the deletion end position in the combined window is updated to match that of w_{i+k}.

1.13. Additional filters during joint calling

PopDel applies additional filters on the final variant calls to improve the precision of the results. The first filter is applied during the iterative parameter estimation. If in a window a sample (over all read groups) has a coverage below two, no deletion will be initialized from the data of this sample and PopDel will not attempt to genotype the sample later on in the respective window. We assume that this window is unreliable if more than 90% of the samples in the window are lacking coverage and filter the window accordingly. In the case of too high coverage (default at least 100 read pairs of a single read group overlapping one window), the read pairs of this read group are ignored in all calculations. Samples consisting solely of read groups with high coverage in one window will also not be assigned genotypes for the respective window. Another filter, the *relative window coverage,* is the value obtained by dividing the product of window length and number of combined windows m by the estimated deletion length. By default, PopDel requires

$$\frac{m \cdot w}{l} \geq 0.5 \tag{14}$$

Lastly, the variant is filtered if all samples are genotyped as non-carriers.

Supplementary Note 2: Evaluation on simulated and real data

To assess the performance of PopDel, Delly[1], Lumpy[2], Manta[3] and GRIDSS[4] the tools were compared on the different simulated and real data sets described in the Methods. A more detailed description of the setup of the callers and the evaluation is described in the following sections.

2.1. Simulated data with random deletions

The simulation of random variants was performed as described in the Methods. Scripts for the reproduction of the workflow can be found in the GitHub repository (see URL's of the Main text). The setup of the callers is described in the following sections.

2.1.1. Running PopDel on simulated data with random deletions

The PopDel workflow for all tests consisted of the following steps

1. Apply `popdel profile` on each sample individually
2. Apply `popdel call` jointly on all profiles

The option `–r chr21` for limiting the processing to chromosome 21 was applied. No further filtering of the calls was performed.

2.1.2. Running Delly on simulated data with random deletions

Delly was applied jointly on all simulated samples using default parameters, except for the `-n` option for disabling the small indel realignment. All non-deletion variants were removed prior to evaluation. No filtering for "QUAL==PASS" was applied, because doing so had a negative impact on the performance of Delly on the simulated data.

2.1.3. Running Lumpy on simulated data with random deletions

Lumpy was applied via Smoove, as recommended by the authors. The recommended file for excluding regions on GRCh37/GRCh38 was used. Additionally, all mappings to chromosomes other than chromosome 21 were ignored in the random deletion simulation by using the expression
`'chr1,chr2,chr3,chr4,chr5,chr6,chr7,chr8,chr9,chr10,chr11,chr12,chr13,chr14,chr15,chr16,chr17,chr18,chr19,chr20,chr22,chrX,chrY,chrM,chrEBV,~.*_.*'`
with the option `--excludeChroms`. The Smoove pipeline consisted of the following steps:

1. Single sample calling and genotyping with `smoove call` for each sample individually (`--processes 1`)
2. Merging the calls of all samples with `smoove merge`
3. Genotyping of each individual sample against the merged calls with `smoove genotype`.
4. Joining the calls of all samples using `smoove paste`.

All non-deletion variants were removed prior to evaluation. No further filtering was performed.

2.1.4. Running Manta on simulated data with random deletions

Manta's workflow consisted of two steps:

1. Configuration via `configManta.py` for all bam files together (`--region chr21`)
2. Joint calling via `runWorklow.py` with `-m local –j 1` (local machine and one thread)

All non-deletion variants were removed prior to evaluation. No filtering for "QUAL==PASS" was performed, because doing so had a negative impact on the performance of Manta on the simulated data. Because the additional variants in the "candidate" file had a very high false positive rate, they were not added to the final output.

2.1.5. Running GRIDSS on simulated data with random deletions

GRIDSS was applied on all samples jointly with an 8 GB heap size for the JVM and one thread. Variants with "FILTER==LOW_QUAL" were removed. An adapted version of the annotation script provided in GRIDSS' GitHub repository [https://github.com/PapenfussLab/gridss/blob/master/example/simple-event-annotation.R] was used to convert the break points into deletion calls.

2.1.6. Evaluation on simulated data with random deletions

A variant called by one of the tools was considered to be a true positive (TP) if `bedtools intersect`[5] reported a reciprocal overlap of least 50% between the area spanned by the called variant and the area spanned by some true simulated variant. All simulated variants in the truth set that hat no such overlap were counted as false negatives (FN). If no such overlap existed, it was considered a false positive (FP). Each simulated variant was only allowed to be matched with one predicted variant. Genotypes of different samples were not considered in this evaluation.

2.1.7. Alternative evaluation with genotype consideration

In addition to the above approach, we applied an alternative evaluation metric that also takes the genotypes of the different samples into account. A called variant was considered to match a simulated true variant if the start position of the call was within 300 bp of the simulated variant's start position and the predicted length of the call was within 150 bp of the real deletion length. Further, the genotypes were inspected. If the predicted genotype of a sample was equal to the simulated genotype, it was counted as two correct alleles. If the predicted genotype did not match the simulated genotype and both genotypes were homozygous, two incorrect alleles were counted. If the predicted genotype did not match the simulated genotype and one of the genotypes was heterozygous, one correct and one incorrect allele were counted. If a simulated variant had no matching predicted variant, incorrect alleles were counted accordingly. Predicted 0/0 genotypes were not counted as correct alleles, because otherwise a tool could arbitrarily boost its score by calling an alleged variant that was not present in the truth set and giving all 0/0 genotypes. The resulting plots of this approach are displayed in Supplementary Figure 8. Because GRIDSS does not produce genotypes, it was excluded from the genotype evaluation.

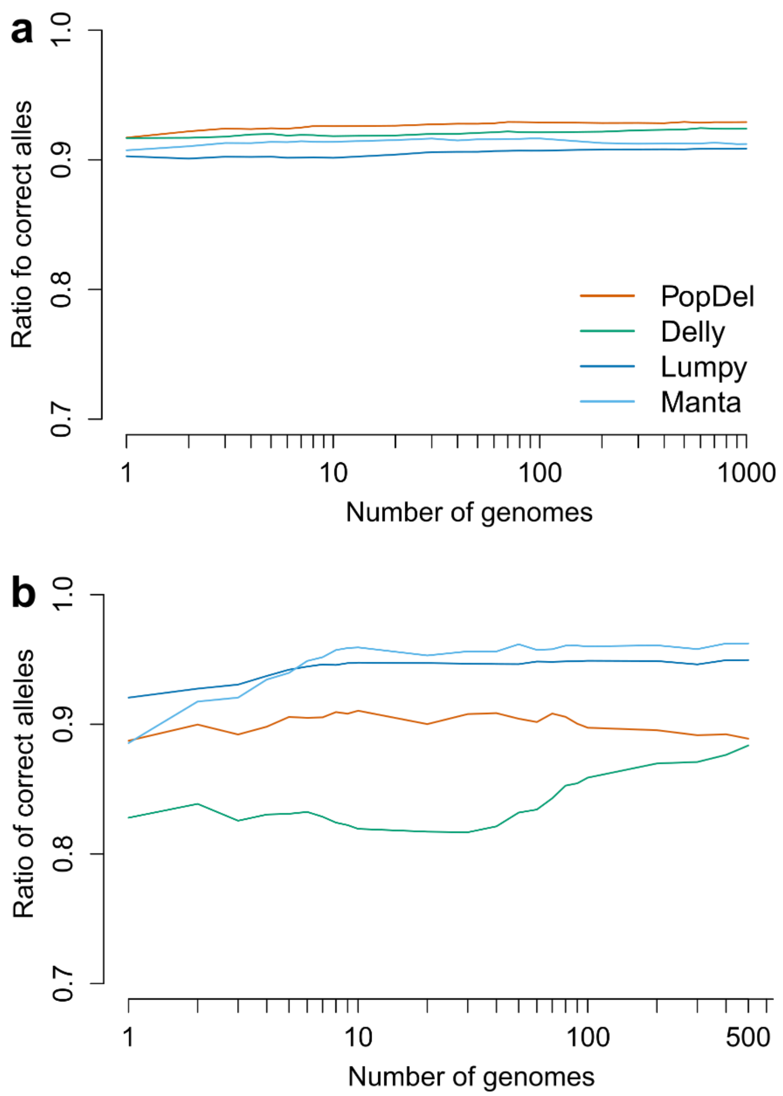

Supplementary Figure 8 – Genotyping evaluation based on alternative overlap evaluation. The approach applied thresholds for the maximum position difference of 300 bp and threshold for the maximum deletion length estimate of 150 bp. Results are shown for **a** random deletions on chromosome 21 and **b** G1k deletions on chromosomes 17 to 22. All genotyped alleles were counted and compared to the simulated haplotypes. Any incorrect allele was counted as an error.

2.1.8. Assessment of running time and memory consumption

Resource consumption of all tools for all tests on simulated data was measured using `/usr/bin/time`. CPU time was calculated as the sum of CPU seconds the process spent in user mode (%U) and the number of CPU seconds the process spent in kernel mode (%S). Memory consumption was measured as maximum resident set size of the process during its lifetime (%M). Wall clock time was measure as the elapsed time (%e).

2.1.9. Tradeoff between running time and memory consumption for PopDel

PopDel offers the option for individually setting the maximum number of buffered windows. This allows managing the memory consumption of PopDel while also influencing the running time in a direct manner. For measuring the effect of varying buffer sizes on running time and memory consumption, PopDel was applied with varying settings for the buffer size on 100 samples simulated from chromosome 21 with random deletions. The results are displayed in Supplementary Figure 9 below.

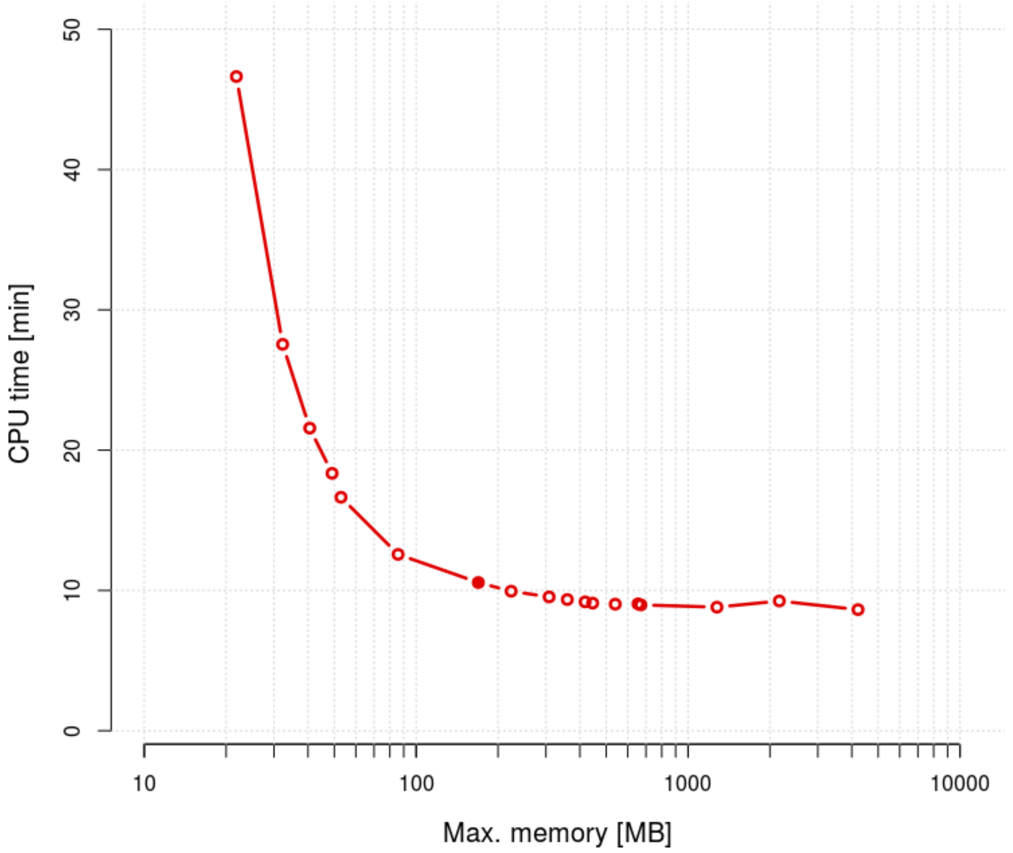

Supplementary Figure 9 – PopDel's resource consumption for varying buffer sizes. PopDel call memory consumption compared to its CPU time for varying buffer sizes on 100 samples simulated from chromosome 21 with random deletions. The filled red dot indicates the default option for the buffer size (200,000 windows).

2.2. Simulated 1000 Genomes Project data

The simulation of variants taken from the 1000 Genomes Project[6] was performed as described in the Methods. Scripts for the reproduction of the workflow can be found in the GitHub repository (see Data availability section of the Main text). The setup of the callers was the same as described in sections 2.1.1-2.1.5, with the exception that chromosomes 17 to 22 were considered. The same evaluation criteria as described in 2.1.6 were applied.

2.3. Evaluation of single and trio wise calling using NA12878 and the HG002 trio

In a first comparison of the performance on real data, we evaluated the precision and recall and the call set overlaps between the tools' predictions and reference deletion sets for NA12878, HG002. The accession numbers, procedure of the mapping, setup of the callers and evaluation metrics are described in the following sections.

2.3.1. Accession numbers for HG002 trio data

Because the HG002 trio was sequenced to a very deep coverage, we decided to use only read data from the first three flow cells of the GIAB data repository. The resulting alignments for HG002, HG003 and HG004 reached an average coverage of 45x, 68x and 72x, respectively. We used the FASTQ files that are deposited in the European Nucleotide Archive (https://www.ebi.ac.uk/ena) with the following accession numbers:

HG002:

SRR1766443, SRR1766444, SRR1766445, SRR1766455, SRR1766456, SRR1766457, SRR1766467, SRR1766468, SRR1766469, SRR1766479, SRR1766480, SRR1766481, SRR1766491, SRR1766492, SRR1766493, SRR1766503, SRR1766504, SRR1766505, SRR1766515, SRR1766516, SRR1766517, SRR1766527, SRR1766528, SRR1766529, SRR1766539, SRR1766540, SRR1766541, SRR1766555, SRR1766556, SRR1766557, SRR1766566, SRR1766567, SRR1766568, SRR1766582, SRR1766583, SRR1766584

HG003:

SRR1766542, SRR1766543, SRR1766545, SRR1766546, SRR1766576, SRR1766577, SRR1766579, SRR1766580, SRR1766589, SRR1766590, SRR1766592, SRR1766593, SRR1766596, SRR1766597, SRR1766599, SRR1766601, SRR1766602, SRR1766603, SRR1766615, SRR1766627, SRR1766639, SRR1766651, SRR1766663, SRR1766675, SRR1766687, SRR1766699, SRR1766700, SRR1766701, SRR1766713, SRR1766714, SRR1766726, SRR1766727, SRR1766728, SRR1766740, SRR1766741, SRR1766742

HG004:

SRR1766763, SRR1766764, SRR1766765, SRR1766775, SRR1766776, SRR1766777, SRR1766787, SRR1766788, SRR1766789, SRR1766799, SRR1766800, SRR1766801, SRR1766811, SRR1766812, SRR1766813, SRR1766823, SRR1766824, SRR1766825, SRR1766835, SRR1766836, SRR1766837, SRR1766847, SRR1766848, SRR1766849,

SRR1766850, SRR1766860, SRR1766861, SRR1766871, SRR1766872, SRR1766873, SRR1766883, SRR1766884, SRR1766885, SRR1766895, SRR1766896, SRR1766897

2.3.2. Mapping of real data

Reads of NA12878 were mapped to GRCh38 and reads of HG002 and his parents to GRCh37 using `bwa mem` with its '`-M`' and '`-R`' options. SAM to BAM conversion, sorting and indexing was performed using Samtools[7]. Duplicates were marked using Picard tools[8].

2.3.3. Running PopDel on NA12878 and the HG002 trio

Creation of the profiles was performed using the default options of `popdel profile`. For NA12878, calling was performed in parallel on chromosomes 1 to 22 and the X chromosome using the option '`-r`'. For the HG002 trio, calling was performed in parallel on chromosomes 1 to 22 using the option '`-r`'. As the HG002 trio has a quite high coverage, the maximum coverage for PopDel to consider was set to the three-fold mean coverage of each sample using the option '`-A`'.

Example command line:

```
popdel profile sample.bam;
popdel call profile_paths.txt -r chr1 -o sample.chr1.vcf;
```

2.3.4. Running Delly on NA12878 and the HG002 trio

Calling was performed using Delly's recommended options. For NA12878, the recommended file of excluded regions on GRCh38 was used and only chromosomes 1 to 22 and the X chromosome were considered by adding the Y chromosome to the file of excluded regions. The HG002 trio was analyzed with the recommended file of excluded regions on GRCh37.

Example command line:

```
delly call -n -x human.hg38.excl.tsv -o sample.bcf -g ref.fasta sample.bam;
```

2.3.5. Running Lumpy on NA12878 and the HG002 trio

Calling was performed using the recommended options of Smoove. For NA12878, the recommended file of excluded regions on GRCh38 was used and only mappings to chromosomes 1 to 22 and the X chromosome were considered. For the HG002 trio, the recommended file of excluded regions on GRCh37 was used and only mappings to chromosomes 1 to 22 were considered.

Example command line:

```
smoove call -x −−outdir sample.out −−name sample.name \
```

```
--exclude exclude.cnvnator_100bp.GRCh38.20170403.bed --excludeChroms \
'chrY,chrM,chrEBV,~.*_.*' --fasta ref.fasta -p 1 --genotype sample.bam;
```

2.3.6. Running Manta on NA12878 and the HG002 trio

Calling was performed using Manta's recommended options. For NA12878 the calling was limited to chromosomes 1 to 22 and the X chromosome.

Example command line:

```
configManta.py --bam sample.bam --referenceFasta ref.fa \
--runDir workingdir --callRegions regions.bed.gz;
workingdir/runWorkflow.py -m local -j 1;
```

2.3.7. Evaluation using `bedtools intersect`

Called variants and the reference deletion sets were converted to BED format and filtered for the desired length range (500-10,000 bp). Any variants with any overlap with a centromeric region (determined by `bedtools intersect`) were removed. Subsequently, overlaps were generated in a hierarchical fashion using Snakemake[9] and BEDtools, as displayed in Supplementary Figure 10.

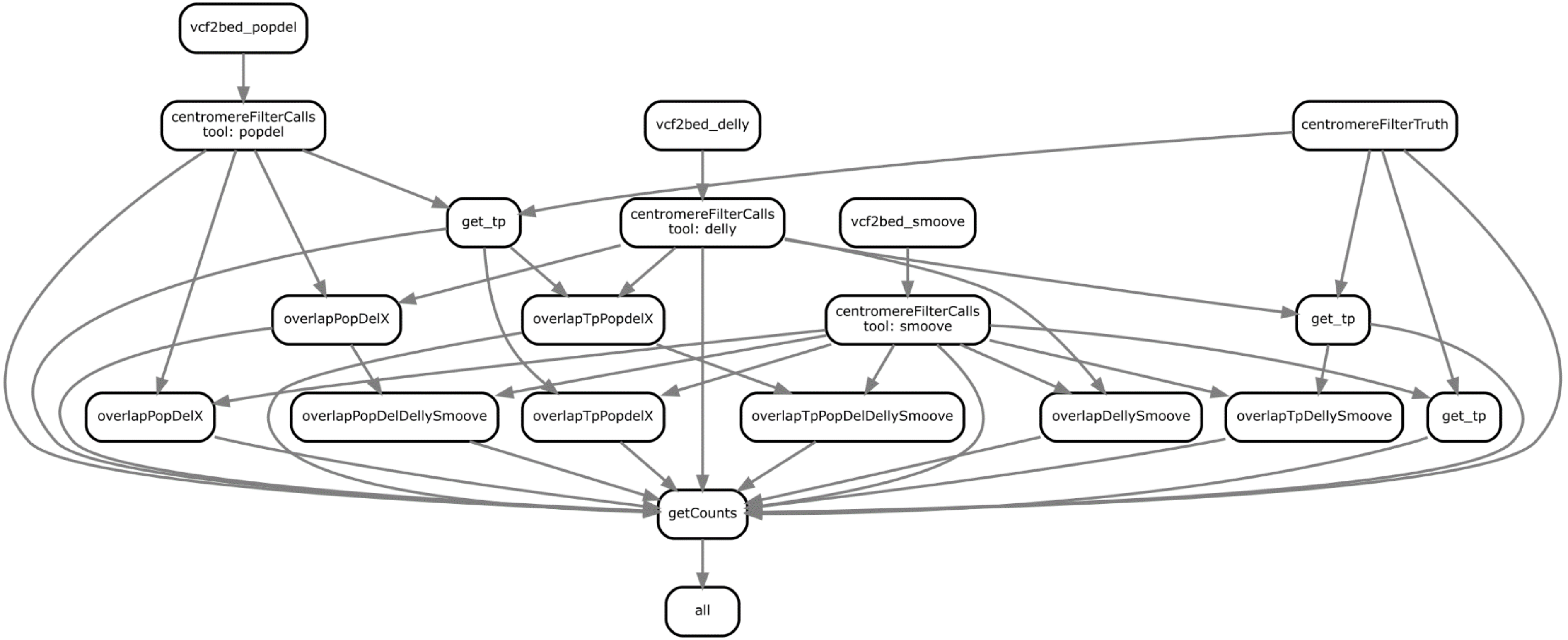

Supplementary Figure 10 - Overview of the overlap generation workflow. The directed graph visualizes the working steps of Snakemake for the computation of the call set overlaps.

2.3.8. Analysis of the Mendelian inheritance error rate

Similar to the approach described for the Polaris Kids cohort in the Main text and below in section 2.4.5, we assessed the Mendelian inheritance error rate on the data of the HG002 trio when filtering for increasing genotype quality (GQ) thresholds (see Supplementary Figure 11). Due to the low number of variants in this single trio, we observe large fluctuation in the error rates

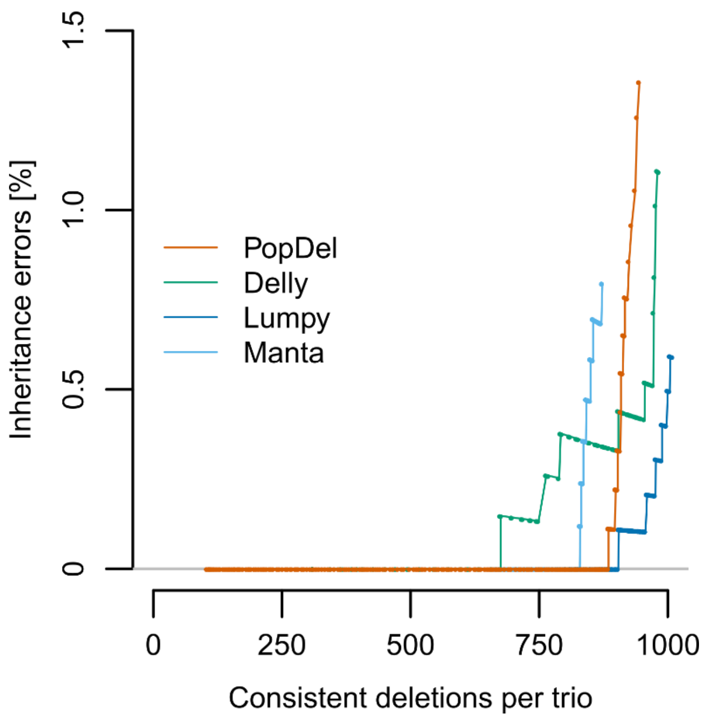

Supplementary Figure 11 - Mendelian inheritance error rate on HG002 trio. The x-axis indicates the number of deletion sites in the HG002 trio that are consistent with Mendelian inheritance; the y-axis indicates the percentage of Mendelian inheritance errors. The grey line indicates perfect 0% inheritance error.

2.4. Evaluation on the Polaris Diversity and Kids cohort

We applied PopDel, Delly and Lumpy on all 150 samples of the Polaris Diversity cohort and 49 trios of the Polaris Kids cohort as described in the following sections. As we could not produce a workflow for Manta that finished on that many whole genomes, we do not provide a workflow here.

2.4.1. Running PopDel on the Polaris Diversity and Kids cohort

The same default approach as described for NA12878 in section 2.3.3 was applied. Chromosomes 1 to 22 and the X chromosome were considered for the Diversity cohort and chromosomes 1 to 22 were considered for the Kids cohort.

2.4.2. Running Delly on the Polaris Diversity and Kids cohort

As jointly calling all 150 samples of the Diversity cohort or 49 trios of the Kids cohort did not produce any results within 4 weeks (option '`-n`' enabled), a sample-wise approach had to be chosen for Delly. It was comprised of the following steps:

1. Sample-wise calling (`delly -n`)
2. Filter all non-deletion variants (`bcftools filter`)
3. Merge all variants of all samples (`delly merge`)
4. Sample-wise genotyping against the list of merged variants (`delly genotype`)
5. Filter variants with invalid positions (`bcftools filter`)
6. Sort and index filtered variants
7. Merge the genotyped calls of all samples (`bcftools merge`)
8. Index the BCF file of all genotyped variants
9. Apply germline filter (`delly filter`)

Steps 5 and 6 were necessary due to a known bug in Delly [https://github.com/dellytools/delly/issues/106] causing some variants to be have POS==0. This made the resulting BCF files impossible to sort and index with BCFtools, which was necessary for merging and applying the germline filter.

2.4.3. Running Lumpy on the Polaris Diversity and Kids cohort

The same default approach as for the simulated data sets (section 2.1.3) was applied, except that chromosomes 1 to 22 and the X chromosome were considered for the Diversity cohort and chromosomes 1 to 22 for the Kids cohort. Further, all non-deletion variants were removed from the call sets after single-sample calling with `smoove call`.

2.4.4. Principal Component Analysis (PCA)

The variants were converted into a sample by variant count matrix M. Field $\mathrm{M}_{i,j} \in \{0,1,2\}$ contains the number of variant alleles sample i carries for variant j. Duplicate rows were removed, as they were uninformative for the PCA. Linkage disequilibrium (LD) was accounted for by calculating the correlation of neighboring variants and removing those with a significant Spearman correlation coefficient (P value threshold 0.05). The procedure was applied iteratively until no more significant correlations were found. From initially 12,203 variants, 4,339 were removed because they had the same allele counts as another variant for all samples (duplicate rows). 3 more variants were removed because they had the same counts for all samples. LD filtering removed 1,589 additional variants, leaving 6,272 variants (48.6%) for the final PCA. PCA was computed using the R-function `prcomp` with the options for 0-centering

and unit variance scaling of the variables enabled. PCA was also performed for the genotypes produced by Delly and Lumpy producing similar results (Supplementary Figure 3).

2.4.5. Genotype quality filtering for Mendelian error rate and transmission rate

For assessing the effect of filtering on the Mendelian inheritance error rate, we applied an increasing genotype quality (GQ) threshold. Further, all deletions that were not in Hardy-Weinberg equilibrium (P value threshold 0.01) were removed. All genotypes below the required GQ were excluded. Because most tools calculate the GQ in a different way, we determined at which point the Mendelian inheritance error rate of the tools dropped below 0.3% to calibrate the filter for the tools in a fair fashion. The GQ values for the tools were 26 (PopDel), 28 (Delly) and 78 (Lumpy). Those filter settings were also used during the calculation of the transmission rates (Table 1).

2.4.6. Transmission rate pattern observed for Lumpy

The deletion genotypes predicted by Lumpy in the Polaris Kids cohort do not reach the expected transmission rate of 50% in our analysis (Figure 5) but stay below 48.3% independent of the genotype quality threshold we apply. A transmission rate below 50% can be explained by two scenarios:

1. False negative calls in the child when a heterozygous genotype was correctly called in one parent.
2. False positive calls in one of the parents when the child was correctly determined as a non-carrier.

We think that false positive calls in one of the parents are more likely than genotyping errors in the child when one parent is correctly called heterozygous.

2.5. Evaluation on Icelandic data

We evaluated PopDel on different datasets of deCODE Genetics at several stages of PopDel’s development.

2.5.1. Transmission and Mendelian inheritance error on Icelandic data

PopDel v1.0.6 was applied on WGS data of 6,794 Icelandic trios. For all variants with a genotype quality threshold above 26, the Mendelian inheritance error rate and transmission rate were calculated considering only variants occurring in a single family with one parent being heterozygous for the variant and the other parent carrying only the reference allele.

2.5.2. Sanity checks on Icelandic data

An early version of PopDel (V0.1-alpha-c74a6c0) was applied on WGS data of 4,959 Icelanders to examine the degree of agreement of PopDel's results with expectations based on previous studies and PopDel's behavior on big datasets.

A non-trivial task when performing variant calling on population scale data is the detection of overlapping variants, at potentially low allele frequencies. Supplementary Figure 12 shows an example common deletion completely overlapping a small deletion with a low allele frequency (2 of 9,918 alleles). PopDel could identify and genotype the two variants correctly using joint calling.

The distribution of the number of variants per genome is expected to follow a normal distribution. The variants identified by PopDel follow this distribution as shown in Supplementary Figure 13. The two visible peaks are thought to originate from sequencing technology related properties.

Further, more small deletions are expected to be present than big ones. Supplementary Figure 14 shows that PopDel identifies exponentially more small deletions than big deletions.

A similar behavior is expected and can be observed for the allele frequency, which is shown in Supplementary Figure 15.

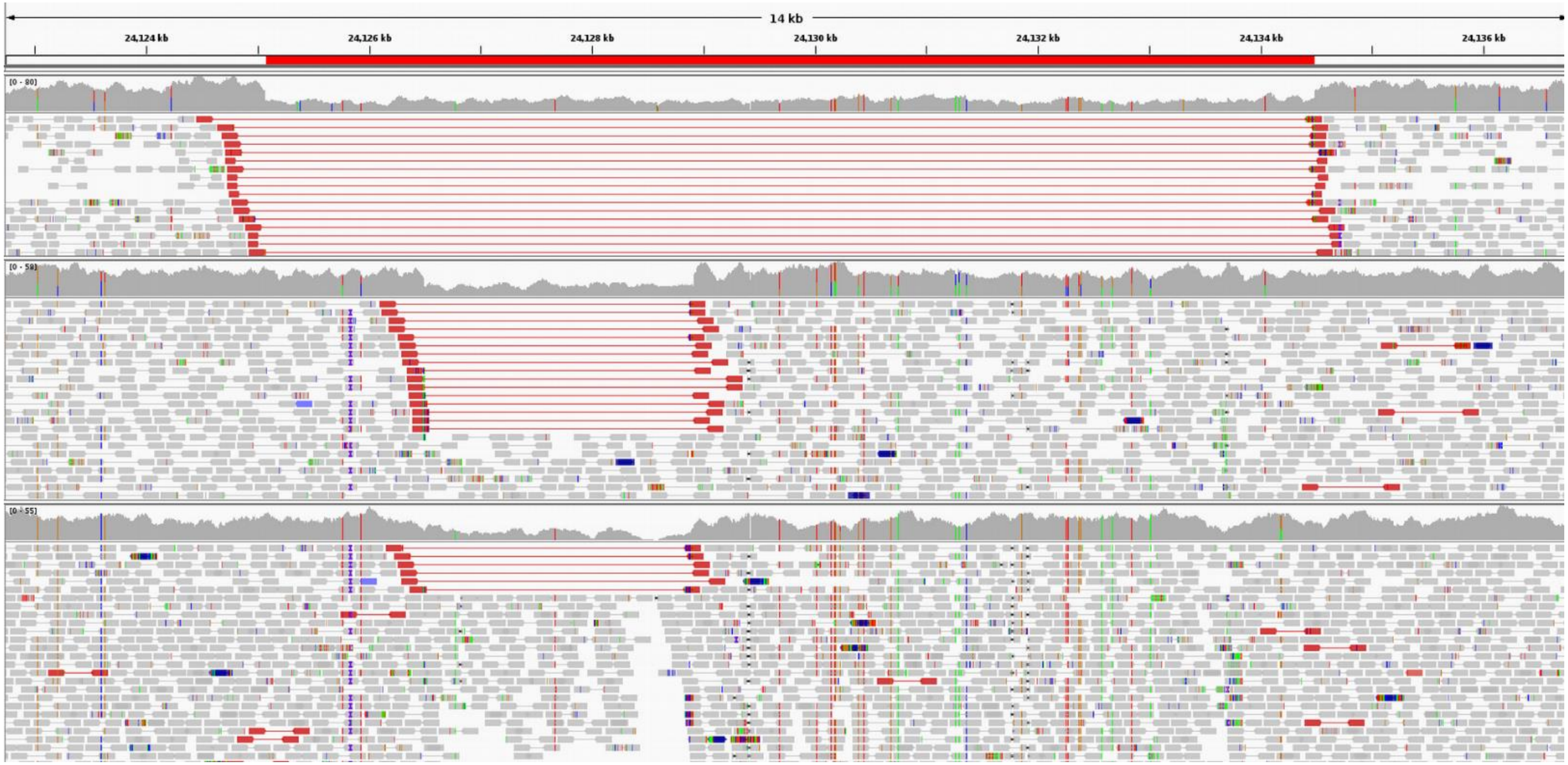

Supplementary Figure 12 – IGV screenshot showing a rare deletion overlapped by a longer common deletion. The smaller deletion (middle and bottom) was only present in two of the analyzed 4,959 genomes, while the longer deletion (top) was present at a much higher allele frequency.

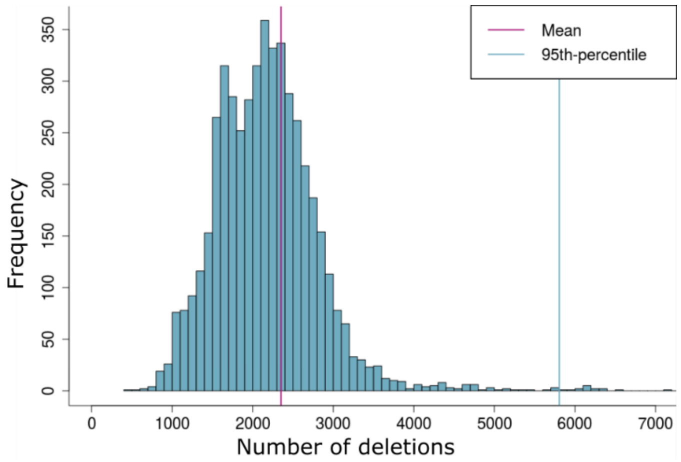

Supplementary Figure 13 – Distribution of deletions per genome as identified jointly by PopDel on 4,959 Icelandic genomes (n=168,101 deletions). The mean was calculated as the arithmetic mean of the sizes of all called variants and the empirical percentiles were used for the 95th percentile.

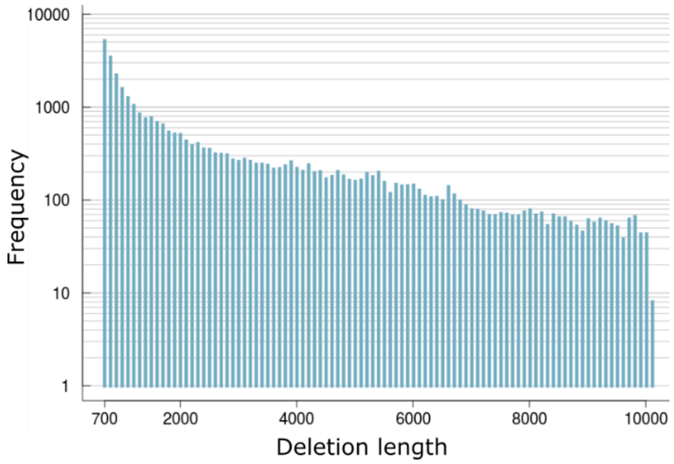

Supplementary Figure 14 – Histogram of deletions lengths identified jointly by PopDel on 4,959 Icelandic genomes (n=168,101 deletions).

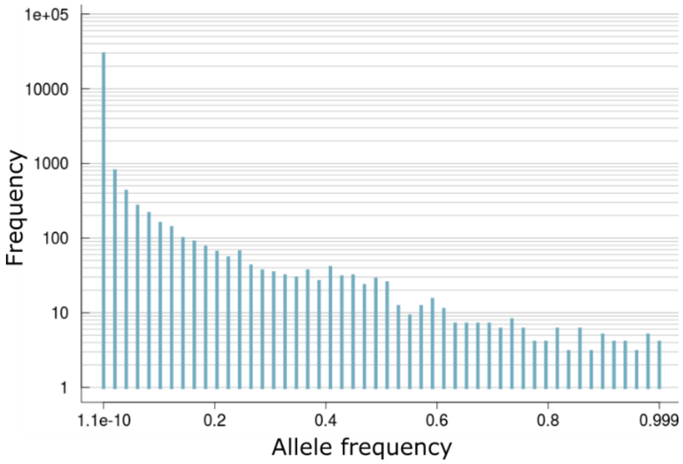

Supplementary Figure 15 – Histogram of allele frequencies of deletions identified jointly by PopDel on 4,959 Icelandic genomes (n=168,101 deletions).

References

1. Rausch, T. *et al.* DELLY: Structural variant discovery by integrated paired-end and split-read analysis. *Bioinformatics* **28**, 333–339 (2012).

2. Layer, R. M., Chiang, C., Quinlan, A. R. & Hall, I. M. LUMPY: a probabilistic framework for structural variant discovery. *Genome Biol.* **15**, R84 (2014).

3. Chen, X. *et al.* Manta: Rapid detection of structural variants and indels for germline and cancer sequencing applications. *Bioinformatics* **32**, 1220–1222 (2016).

4. Cameron, D. *et al.* GRIDSS: sensitive and specific genomic rearrangement detection using positional de Bruijn graph assembly. Submitted for publication to Nature Methods. (2016) doi:10.1101/110387.

5. Quinlan, A. R. & Hall, I. M. BEDTools: A flexible suite of utilities for comparing genomic features. *Bioinformatics* **26**, 841–842 (2010).

6. Consortium, T. 1000 G. P. A global reference for human genetic variation. *Nature* vol. 526 68–74 (2015).

7. Li, H. *et al.* The Sequence Alignment/Map format and SAMtools. *Bioinformatics* **25**, 2078–2079 (2009).

8. Broad Institute. Picard Toolkit. broadinstitute.github.io/picard. (2018).

9. Köster, J. & Rahmann, S. Snakemake-a scalable bioinformatics workflow engine. *Bioinformatics* **28**, 2520–2522 (2012).
