# Supplementary material for: PopDel identifies medium-size deletions simultaneously in tens of thousands of genomes
Source: Nat Commun. 2021 Feb 1;12:730. doi: 10.1038/s41467-020-20850-5 (PMC7851401; doi:10.1038/s41467-020-20850-5)
Supplement: Supplementary file 2 — Reporting Summary [file 41467_2020_20850_MOESM2_ESM.pdf]

nature research

Corresponding author(s): Birte Kehr

Last updated by author(s): Nov 23, 2020

Reporting Summary

Nature Research wishes to improve the reproducibility of the work that we publish. This form provides structure for consistency and transparency in reporting. For further information on Nature Research policies, see our Editorial Policies and the Editorial Policy Checklist.

Statistics

For all statistical analyses, confirm that the following items are present in the figure legend, table legend, main text, or Methods section.

n/a	Confirmed	
☐	☒	The exact sample size (n) for each experimental group/condition, given as a discrete number and unit of measurement
☒	☐	A statement on whether measurements were taken from distinct samples or whether the same sample was measured repeatedly
☐	☒	The statistical test(s) used AND whether they are one- or two-sided<br>*Only common tests should be described solely by name; describe more complex techniques in the Methods section.*
☒	☐	A description of all covariates tested
☒	☐	A description of any assumptions or corrections, such as tests of normality and adjustment for multiple comparisons
☐	☒	A full description of the statistical parameters including central tendency (e.g. means) or other basic estimates (e.g. regression coefficient) AND variation (e.g. standard deviation) or associated estimates of uncertainty (e.g. confidence intervals)
☐	☒	For null hypothesis testing, the test statistic (e.g. F, t, r) with confidence intervals, effect sizes, degrees of freedom and P value noted<br>*Give P values as exact values whenever suitable.*
☒	☐	For Bayesian analysis, information on the choice of priors and Markov chain Monte Carlo settings
☒	☐	For hierarchical and complex designs, identification of the appropriate level for tests and full reporting of outcomes
☒	☐	Estimates of effect sizes (e.g. Cohen's d, Pearson's r), indicating how they were calculated

Our web collection on statistics for biologists contains articles on many of the points above.

Software and code

Policy information about availability of computer code

Data collection	No software was used to collect data
Data analysis	The source code of PopDel (v1.2.2) is available on GitHub: https://github.com/kehrlab/PopDel<br>Scripts used for analysis are available at https://github.com/kehrlab/PopDel-scripts<br><br>Other tools used for data analysis include Delly (0.7.8), GRIDSS (1.8.1), Manta (1.6.0), Smoove (0.2.4 with Lumpy 0.2.13 and SVtyper 0.7.0), art_illumina (2.5.8), BWA-mem(0.7.17-r1188), NCBI Genome Remapping Service, Bcftools (1.9), Samtools (1.9), Bedtools (2.29.0), IGV (2.3), Picard tools (2.21.2), Snakemake (5.7.4), bwakit (0.7.15). Command line parameters of all tools are provided in the Methods and Supplementary material.

For manuscripts utilizing custom algorithms or software that are central to the research but not yet described in published literature, software must be made available to editors and reviewers. We strongly encourage code deposition in a community repository (e.g. GitHub). See the Nature Research guidelines for submitting code & software for further information.

Data

Policy information about availability of data

All manuscripts must include a data availability statement. This statement should provide the following information, where applicable:

- Accession codes, unique identifiers, or web links for publicly available datasets
- A list of figures that have associated raw data
- A description of any restrictions on data availability

The generated VCF files used for evaluation of all simulated and real data sets have been deposited in Zenodo (DOI: 10.5281/zenodo.3992607). The deletion set of the 1000 Genomes Project we used for simulating data was obtained from ftp://ftp.1000genomes.ebi.ac.uk/vol1/ftp/phase3/integrated_sv_map/

ALL.wgs.mergedSV.v8.20130502.svs.genotypes.vcf.gz.
The short read data from the HG001/NA12878 genome is publicly available under ERA run accession ERR194147 [https://www.ebi.ac.uk/ena/browser/view/ERR194147]. The short read data from the HG002/NA24385 genome and parental genomes HG003/NA24149 and HG004/NA24143 are available under BioProject accession PRJNA200694 [https://www.ebi.ac.uk/ena/browser/view/PRJNA200694]. The precise list of run accession numbers we used for HG002 and his parents is given in the Supplementary Note 2.
The GIAB short read reference set for HG001/NA12878 was obtained from ftp://ftp-trace.ncbi.nlm.nih.gov/giab/ftp/technical/svclassify_Manuscript/Supplementary_Information/Personalis_1000_Genomes_deduplicated_deletions.bed and the long read reference call sets from ftp://ftp-trace.ncbi.nlm.nih.gov/giab/ftp/data/NA12878/NA12878_PacBio_MtSinai/NA12878.sorted.vcf.gz. The preliminary variant set for HG002 is available at ftp://ftp-trace.ncbi.nlm.nih.gov/giab/ftp/data/AshkenazimTrio/analysis/NIST_SVs_Integration_v0.6/HG002_SVs_Tier1_v0.6.vcf.gz and the high-confidence regions for HG002 at ftp://ftp-trace.ncbi.nlm.nih.gov/giab/ftp/data/AshkenazimTrio/analysis/NIST_SVs_Integration_v0.6/HG002_SVs_Tier1_v0.6.bed.
The short read data of the Polaris HiSeq X Diversity cohort is publicly available under BioProject accession PRJEB20654 [https://www.ebi.ac.uk/ena/browser/view/PRJEB20654] and the Polaris HiSeq X Kids cohort under BioProject accession PRJEB25009 [https://www.ebi.ac.uk/ena/browser/view/PRJEB25009].
We obtained the human reference genome GRCh37 from ftp://ftp.1000genomes.ebi.ac.uk/vol1/ftp/technical/reference/phase2_reference_assembly_sequence/hs37d5.fa.gz and downloaded GRCh38 on June 14th, 2017 from ftp://ftp.ncbi.nlm.nih.gov/genomes/all/GCA/000/001/405/GCA_000001405.15_GRCh38/seqs_for_alignment_pipelines.ucsc_ids/GCA_000001405.15_GRCh38_full_analysis_set.fna.gz using bwakit (0.7.15).
Access to raw Icelandic sequence data is restricted by Icelandic state law and can be given through collaboration with KS.

Field-specific reporting

Please select the one below that is the best fit for your research. If you are not sure, read the appropriate sections before making your selection.

- [x] Life sciences
- [ ] Behavioural & social sciences
- [ ] Ecological, evolutionary & environmental sciences

For a reference copy of the document with all sections, see nature.com/documents/nr-reporting-summary-flat.pdf

Life sciences study design

All studies must disclose on these points even when the disclosure is negative.

Sample size	Previously published datasets were used, no sample size was selected. Simulation was carried out on a range of 1 to 1000 samples. The data sets were selected to cover different evaluation aspects, i.e. simulated data with ground truth, public benchmark data with reference sets, trio data for analysis of inheritance patterns, and Icelandic data to test scalability.
Data exclusions	Due to unavailability of the data of one Polaris offspring (ERR2304561), this sample and corresponding parents had to be excluded from the analysis of the Polaris trios.
Replication	Not applicable since the study focus is a new computational method. A variety of different data sets each of considerable size was used to assess the performance of the new method in various ways.
Randomization	Count matrix of variants identified in the Polaris Diversity cohort were shuffled before PCA.
Blinding	Not applicable as no subjective evaluations were performed.

Reporting for specific materials, systems and methods

We require information from authors about some types of materials, experimental systems and methods used in many studies. Here, indicate whether each material, system or method listed is relevant to your study. If you are not sure if a list item applies to your research, read the appropriate section before selecting a response.

Materials & experimental systems

n/a	Involved in the study	
x		Antibodies
x		Eukaryotic cell lines
x		Palaeontology and archaeology
x		Animals and other organisms
	x	Human research participants
x		Clinical data
x		Dual use research of concern

Methods

n/a	Involved in the study	
x		ChIP-seq
x		Flow cytometry
x		MRI-based neuroimaging

Human research participants

Policy information about studies involving human research participants

Population characteristics	See Jónsson, H., Sulem, P., Kehr, B. et al. Whole genome characterization of sequence diversity of 15,220 Icelanders. Sci Data 4, 170115 (2017). https://doi.org/10.1038/sdata.2017.115
Recruitment	See Jónsson, H., Sulem, P., Kehr, B. et al. Whole genome characterization of sequence diversity of 15,220 Icelanders. Sci Data 4, 170115 (2017). https://doi.org/10.1038/sdata.2017.115
Ethics oversight	National Bioethics Committee and Data Protection Authority in Iceland

Note that full information on the approval of the study protocol must also be provided in the manuscript.
